# Vaccination Week in the Americas, 2011: an opportunity to assess the routine vaccination program in the Bolivarian Republic of Venezuela

**DOI:** 10.1186/s12889-015-1723-4

**Published:** 2015-04-17

**Authors:** Daniel Sánchez, Samir V Sodha, Hannah J Kurtis, Gladys Ghisays, Kathleen A Wannemuehler, M Carolina Danovaro-Holliday, Alba María Ropero-Álvarez

**Affiliations:** Programa Nacional de Inmunizaciones de Venezuela, Ministerio del Poder Popular para la Salud (MPPS), Caracas, Bolivarian Republic of Venezuela; Centers for Disease Control and Prevention, Atlanta, GA 30329-4027 USA; Comprehensive Family Immunization Unit, Pan American Health Organization, 525 23rd St NW, Washington DC, 20037-2895 USA; Pan American Health Organization Country Office, Caracas, Bolivarian Republic of Venezuela; Current address: Pan American Health Organization Country Office, Quito, Ecuador

**Keywords:** Vaccination Week in the Americas, Expanded Program on Immunization, Cluster survey, Venezuela

## Abstract

**Background:**

Vaccination Week in the Americas (VWA) is an annual initiative in countries and territories of the Americas every April to highlight the work of national expanded programs on immunization (EPI) and increase access to vaccination services for high-risk population groups. In 2011, as part of VWA, Venezuela targeted children aged less than 6 years in 25 priority border municipalities using social mobilization to increase institution-based vaccination. Implementation of social communication activities was decentralized to the local level. We conducted a survey in one border municipality of Venezuela to evaluate the outcome of VWA 2011 and provide a snapshot of the overall performance of the routine EPI at that level.

**Methods:**

We conducted a coverage survey, using stratified cluster sampling, in the Venezuelan municipality of Bolivar (bordering Colombia) in August 2011. We collected information for children aged <6 years through caregiver interviews and transcription of vaccination card data. We estimated each child’s eligibility to receive a specific vaccine dose during VWA 2011 and whether or not they were actually vaccinated during VWA activities. We also estimated baseline vaccination coverage, timeliness and 95% confidence intervals (CI), and used chi-square tests to compare coverage across age cohorts, taking into account the sampling design.

**Results:**

We surveyed 839 children from 698 households; 93% of children had a vaccination card. Among households surveyed, 216 (31%) caregivers reported having heard about a vaccination activity during April or May 2011. Of the 528 children eligible to receive a vaccine during VWA, 24% received at least one dose, while 13% received all doses due. Overall, baseline coverage with routine vaccines, as measured by the survey, was >85%, with a few exceptions.

**Conclusion:**

Low levels of VWA awareness among caregivers probably contributed to the limited vaccination of eligible children during the VWA activities in Bolivar in 2011. However, vaccine coverage for most EPI vaccines was high. Additionally, high vaccination card availability and high participation in VWA among those caregivers aware of it in 2011 suggest public trust in the EPI program in the municipality. Health authorities have used survey findings to inform changes to the routine EPI and better VWA implementation in subsequent years.

## Background

The Bolivarian Republic of Venezuela's Expanded Program on Immunization (EPI) strives to protect its citizens against vaccine-preventable diseases (VPDs); however, suboptimal performance has left multiple birth cohorts susceptible to VPDs. Over the past 3 decades, Venezuela has achieved <85% reported coverage with third dose of diphtheria-tetanus-pertussis [DTP3] vaccine, the common indicator for immunization program performance [1]. Groups at risk for suboptimal immunization coverage include indigenous populations, those in border, urban and hard-to-reach zones, displaced populations and those in areas where population growth exceeds the capacity of health services.

Vaccination Week in the Americas (VWA) is an annual initiative carried out by countries and territories in the Region of the Americas and supported by the Pan American Health Organization/World Health Organization (PAHO/WHO) [2,3]. VWA began in 2003 based on a proposal by ministers of health of six countries^a^ in response to a large measles outbreak in Venezuela and Colombia in 2002 [4]; the proposal called for a coordinated annual international vaccination effort. The initiative’s objectives include highlighting the work of national immunization programs and increasing access to vaccination services, particularly for hard-to-reach, high-risk groups. Specific campaigns undertaken as part of VWA are flexible and chosen by countries based on their public health priorities [5]. VWA’s success has inspired establishment of similar initiatives in other regions, leading to the 2012 launch of World Immunization Week, endorsed by the World Health Assembly [6].

In Venezuela, VWA is a comprehensive initiative encompassing multiple activities including advocacy with political and technical authorities, social communication and mobilization and increased availability of vaccination services. These activities are intended to help position EPI on the political agenda, increase visibility of the program, and improve coverage [7]. The VWA 2011 campaign in Venezuela included targeting children aged less than 6 years of age who were eligible for one or more vaccine doses in the national schedule (Table 1), with emphasis on those who lived in one of 25 high risk border municipalities, selected based on reported low coverage the year prior. The main strategy was increased social mobilization for institution-based vaccinations. Across the region, VWA 2011 was officially scheduled from April 23^rd^ to April 30^th^, but in Venezuela it extended through the month of May.

**Table 1:** **Routine vaccination schedule in Venezuela, 2011**

**Vaccine**	**Recommended age(s)**	**Year of introduction**
BCG^1^	Birth	1940s
Hepatitis B (1^st^ dose)	Birth	2000
Yellow fever	1 year	2000
OPV^2^		
- Primary series	2 m, 4 m, 6 m	1960s
- Booster	18 m, 5 years	2009
Pentavalent^3^		2004
- Primary series	2 m, 4 m, 6 m	
- Pentavalent4 (1^st^ DTP Booster)	18 m	
Rotavirus	2 m, 4 m	2006 (modified 2009)
DTP^4^		
- 2^nd^ DTP booster	5 years	2009
MMR^5^	1 year, 5 years	MMR 1 (1998); MMR 2 (2009)
Td^6^	10 years, women of childbearing age, adults	2009 (replaced TT, used since the 1950s)
Influenza (annual)	6-23 months	2006
	>60 years	
	High-risk groups	
23-valent pneumococcal	>60 years	2008

m = months.

^1^ *BCG = Bacillus Calmette-Guérin.*

^2^ *OPV = oral poliovirus vaccine.*

^3^ *Pentavalent = diphtheria and tetanus toxoids, and pertussis, Haemophilus influenzae type b and hepatitis B vaccines.*

^4^ *DTP = diphtheria and tetanus toxoids and pertussis vaccine.*

^5^ *MMR = measles-mumps-rubella vaccine.*

^6^ *Td = Tetanus and diphtheria toxoids for older children and adults.*

Bolivar is one of 29 municipalities in the western Andean state of Táchira and was one of the prioritized areas during VWA 2011. Bolivar is located on Venezuela’s border with the Republic of Colombia and experiences constant population movement due to commercial activities between the two countries; in 2011, the population of Bolivar was 74,568 individuals [8]. Because the municipality contains urban, periurban, and rural areas, as well as displaced populations, the Venezuelan Ministry of Health (MOH) selected Bolivar as the site for an evaluation of VWA 2011, including a post-VWA immunization coverage survey [9]. This coverage survey was supported by PAHO and the United States Centers for Disease Control and Prevention (CDC) and represents one of the few methodologically rigorous evaluations performed over the 12 years of VWA.

## Methods

### Target area and population

Our coverage survey was conducted on August 13–14, 2011. For MOH and PAHO, it was considered an operational study and not research. The protocol was reviewed by the human subjects coordinator for CDC’s Center for Global Health, and was considered a programmatic evaluation and exempt from institutional board review of human subjects research. The survey’s target area included the municipality of Bolivar, subdivided into the areas of San Antonio del Táchira (the municipality’s capital and predominately urban area), Palotal (periurban area) and a rural area consisting of Juan Vicente Gómez and Isaías Medina Angarita parishes. Children were eligible to participate if they were less than 6 years of age during April and May 2011.

### Survey design

We calculated a sample size of 267 children eligible to be vaccinated during VWA 2011 assuming 50% of eligible children would receive at least one vaccine, precision of ±6%, and probability of achieving precision of 0.95. We used binomial distribution to calculate a sample size of 720 children less than 6 years of age, based on the assumption only 40% of target age children would be eligible for at least one vaccine and to ensure 90% probability of identifying the target of 267 eligible children.

We used a stratified 30-cluster survey design, proportionally allocating clusters to the 3 areas based on population size (22 in San Antonio del Táchira, 5 in Palotal, and 3 in the rural area). In each stratum, we selected clusters (barrios) with replacement and probability of selection proportional to size. We used population estimates from MOH and included 24 households with at least one child less than 6 years old in each cluster. We selected an area within the cluster using a 10x10 grid superimposed over a map of the cluster and chose one cell within the grid via simple random sampling. The first house within that cell was also chosen at random and from there, we visited all subsequent households to the right, until 24 households with at least one child less than 6 years of age had been surveyed.

### Data collection

A total of 44 interviewers were trained who were health workers^b^ from Bolivar. Survey teams consisted of two interviewers and one supervisor who accompanied two or three teams. Survey teams were assigned to areas outside of their normal jurisdiction and collected data on all children in the target age range within a participating household.

Survey teams administered a standardized questionnaire to any available caregiver (e.g. mother, father, grandmother, etc.), to collect information about the caregiver’s education, household demographics, type of health center routinely visited, awareness about a vaccination activity conducted in their community during April or May 2011, and, when applicable, the caregiver’s actions in response to that knowledge. Survey teams transcribed information in each child’s vaccination card (doses received and dates of administration). When a card was not available, teams reviewed the health center’s vaccination records; teams did not collect caregivers’ recollections about specific vaccine doses received, but did record responses to questions about the campaign and communication. We trained data entry staff who entered data into an EpiInfo® (CDC, Atlanta, GA, USA) database. In the field, accuracy of data entry was reviewed by the technical focal point from the national immunization program.

### Data analysis

We used SAS v9.2 (SAS Institute, Inc., Cary, NC, USA), SUDAAN® v10 (RTI International, Research Triangle Park, NC, USA), and R v3.0 (R Foundation for Statistical Computing, Vienna, Austria) to clean and analyze data. We restricted vaccination-related analyses to children with information available through vaccination cards (and for a small minority, health center records). We determined each child’s eligibility to receive a specific vaccine dose during VWA 2011 based on their date of birth and the information recorded regarding specific doses received. We calculated proportions of children who received any vaccine doses, specific vaccine doses, or all vaccine doses for which they were eligible during VWA 2011 (April 23, 2011 – May 31, 2011). We used all vaccine doses transcribed, regardless of when they were administered, to estimate overall coverage of specific vaccines at the time of the survey, by five 1-year age cohorts. We estimated vaccination coverage, 95% confidence intervals (CI), and Chi-square tests to compare coverage across age cohorts, taking into account the stratification, the first stage cluster sampling, and stratum-level weights (estimated target population in each stratum divided by sample size in each stratum) in SUDAAN. We used R survey package [10] to estimate time to vaccination and 95% CIs (Aalen [hazard-based] estimator) for each dose of pentavalent vaccine^c^ (Penta) and first dose of measles-mumps-rubella vaccine (MMR1). The results are presented in graphs as 1 minus time to vaccination to show estimated change in probability of being vaccinated through 24 months of age (Penta) and 36 months of age (MMR1). To show uncertainty around the estimated curve, 95% CIs for probability of vaccination are calculated and plotted at each time point that an event (i.e., child is vaccinated) occurs.

## Results

### Households surveyed

We surveyed caregivers of 839 children less than six years of age in 698 households, including 513 (74%) households in the urban area of San Antonio del Táchira, 114 (16%) in the semi-urban Palotal, and 71 (10%) in the rural area. Most (84%) were mothers; 8% were grandmothers. Fifty-nine percent of caregivers reported having completed high school or university education. Fifty-two percent of eligible children were female.

Among caregivers surveyed, 90% reported using public outpatient and hospital settings for routine vaccination services, while 5% utilized private institutions and 5% reported other institutions. Among 698 households surveyed, 216 (31%, 95% CI 25-38%) respondents reported having heard about a vaccination activity during April or May 2011. Among these 216, 152 (70%, 95% CI 59-81%) reported having heard about a general campaign, while only 20 (9%, 95% CI 5-18%) identified VWA 2011 specifically. The most common sources of information among those aware of a recent vaccination activity were radio (28%, 95% CI 17-39%) and the local health center (20%, 95% CI 13-29%). When caregivers were asked how they responded after learning about a vaccination activity, 63% (95% CI 54-73%) reported bringing their child to the health center with a vaccination card, 7% (95% CI 2-19%) said they brought their child without a vaccination card, 25% (95% CI 17-34%) indicated that they did not bring their child to the vaccination site and 5% (95% CI 2-10%) of responses were classified as “other”.

### Vaccination during VWA 2011 and EPI coverage

Among 839 surveyed children, 784 (93%) had a vaccination card, and for an additional 6 (1%) children, vaccination data were available from clinic records. Among all 790 children with available vaccination data, 528 (67%, 95% CI 63-71%) were age-eligible to receive at least one vaccine dose during VWA 2011 (Table 2). Among these, 126 (24%) received at least one dose for which they were eligible, and 69 (13%) received all indicated doses. Among the 126 children who received at least one vaccination, 57 (45%) did not receive all vaccinations for which they were eligible. Among 18 children eligible to receive Bacillus Calmette-Guérin vaccine (BCG) and 13 eligible for the first dose of hepatitis B, 16 (89%) and 11 (85%) received BCG and hepatitis B, respectively, representing the largest proportion of eligible children to receive an indicated vaccine dose. By contrast, among children eligible to receive yellow fever vaccine (N = 127), the second booster dose of oral polio vaccine (OPV2) (N = 45), or the second MMR dose (MMR2) (N = 87), only 3 (2%), 1 (2%), and 4 (5%), respectively, received the recommended vaccine(s).

**Table 2:** **Age range of eligibility for receiving vaccine and proportion of eligible children vaccinated during Vaccination Week of Americas, Bolivar, Venezuela, 2011**

**Vaccine**	**Age eligibility (months)**	**Minimum interval (after preceding dose), if applicable**	**No. eligible**	**No. (% of eligible) vaccinated [95% CI]**	
BCG	≤12		18	16	(89)
Hepatitis B	≤1		13	11	(85)
Pentavalent1*	≥2		40	14	(35)
Pentavalent2*	≥4	≥28 days (Penta1)	42	10	(24)
Pentavalent3*	≥6	≥28 days (Penta2)	60	14	(23)
OPV1	≥2		46	23	(50)
OPV2	≥4	≥28 days (OPV1)	52	18	(35)
OPV3	≥6	≥28 days (OPV2)	74	15	(20)
MMR1	≥12		121	16	(13)
Yellow fever	≥12		127	3	(2)
Rotavirus1	2 - 4		20	8	(40)
Rotavirus2	4-8	≥28 days (Rotavirus1)	11	6	(55)
Pentavalent4* (DTP boost1)	≥18		155	14	(9)
DTP boost2	≥60		9	5	(56)
OPV boost1	≥18		138	17	(12)
OPV boost2	≥60		45	1	(2)
MMR2	≥60		87	4	(5)
Any dose	≤72		528	126	(24) [20–28]
All indicated doses	≤72		528	69	(13) [10-16]

*Pentavalent = Diphtheria-Tetanus-Pertussis (DTP) + *Haemophilus influenzae* type b + Hepatitis B vaccines.

### Vaccination coverage

BCG coverage estimates were at least 95% for each 1-year age cohort; however, coverage with birth dose of hepatitis B vaccine was lower, with the lowest being 81% (95% CI 71-88%) in children aged 24–35 months (Table 3).

**Table 3:** **Vaccination coverage ascertained by vaccination card or medical records among 641 surveyed children aged 12–71 months (weighted), by birth cohort, Bolivar, Venezuela, 2011**

**Vaccine dose (recommended age)**	**12-23 months (n = 129)**			**24 – 35 months (n = 131)**			**36 - 47 months (n = 141)**			**48 – 59 months (n = 139)**			**60 - 71 months (n = 101)**		
	**No (%) [95% CI] who received dose**			**No (%) [95% CI] who received dose**			**No (%) [95% CI] who received dose**			**No (%) [95% CI] who received dose**			**No (%) [95% CI] who received dose**		
BCG (birth)	123	(95)	[89–98]	126	(96)	[91–98]	139	(99)	[94- > 99]	136	(98)	[93–99]	99	(98)	[93–99]
Hepatitis B* (birth)	112	(87)	[81–91]	106	(81)	[71–88]	131	(93)	[86–97]	127	(92)	[83–96]	86	(85)	[77–91]
Penta3* (6 months)	117	(91)	[85–94]	121	(92)	[86–96]	127	(90)	[83–94]	137	(99)	[94- >99]	93	(92)	[85–96]
OPV3 (6 months)	111	(86)	[78–91]	121	(92)	[86–96]	126	(89)	[82–94]	133	(96)	[91–98]	93	(92)	[81–97]
MMR1* (12 months)	94	(72)	[63–80]	116	(89)	[81–94]	128	(91)	[79–96]	129	(93)	[85–97]	89	(88)	[77–94]
Yellow fever* (12 months)	81	(62)	[54–70]	107	(82)	[75–87]	130	(92)	[86–96]	132	(95)	[88–98]	94	(93)	[87–97]
Rotavirus2* (4–8 months)	67	(51)	[41–62]	68	(52)	[43–60]	53	(38)	[28–48]	38	(27)	[19–36]	21	(21)	[14–29]
DTP booster1* (18 months)	N/A	N/A	N/A	33	(59)	[52–66]	87	(61)	[48–73]	103	(74)	[62–83]	78	(77)	[67–85]
OPV booster1* (18 months)	N/A	N/A	N/A	84	(64)	[55–72]	91	(64)	[57–75]	113	(81)	[69–90]	80	(79)	[66–88]
DTP booster2 (5 years)	N/A	N/A	N/A	N/A	N/A	N/A	N/A	N/A	N/A	N/A	N/A	N/A	17	(17)	[10–27]
OPV booster2 (5 years)	N/A	N/A	N/A	N/A	N/A	N/A	N/A	N/A	N/A	N/A	N/A	N/A	34	(34)	[22–47]
MMR2 (5 years)	N/A	N/A	N/A	N/A	N/A	N/A	N/A	N/A	N/A	N/A	N/A	N/A	27	(27)	[18–37]

*p value <0.05 indicates a difference in coverage between birth cohorts for given vaccine dose.

Coverage with Penta3 exceeded 90% in most cohorts, and was generally higher than coverage with OPV3, which ranged from 86% in 12–23 month-olds to 96% in 48–59 month-olds. MMR1 coverage was only 72% (95% CI 63-80%) among children aged 12–23 months, although in older cohorts it was approximately 90%. Similarly, yellow fever vaccine coverage estimates were higher (92-95%) in older age cohorts but significantly lower in younger age cohorts, with coverage estimates of 62% (95% CI 54-70%) among children aged 12–23 months and 82% (95% CI 75-87%) in children aged 24–35 months. In contrast, second dose rotavirus vaccine coverage estimates were highest in the two youngest age cohorts (51% [95% CI 41-62%] among 12–23 month-olds and 52% [95% CI 43–60] among 24–35 month-olds), but were significantly lower in the three older age cohorts: among children aged 60–71 months, coverage was only 21% (95% CI 14-29%). Coverage with first DTP and OPV booster doses generally increased with increasing age, from 59% (95% CI 52-66%) (DTP) and 64% (95% CI 55-72%) (OPV) among children aged 24–35 months to 74% (95% CI 62-83%) (DTP) and 81% (95% CI 69-90%) (OPV) among children aged 48–59 months. First booster coverage for children aged 60–71 months continued to increase for DTP at 77% (95% CI 67-85%) but fell slightly for OPV to 79% (95% CI 66-88%). There were statistically significant differences in coverage between birth cohorts for the following vaccine doses: hepatitis B birth dose, Penta3, MMR1, yellow fever vaccine, second rotavirus dose, and first booster doses of DTP and OPV vaccines.

### Timeliness of vaccination

**Figure 1:**
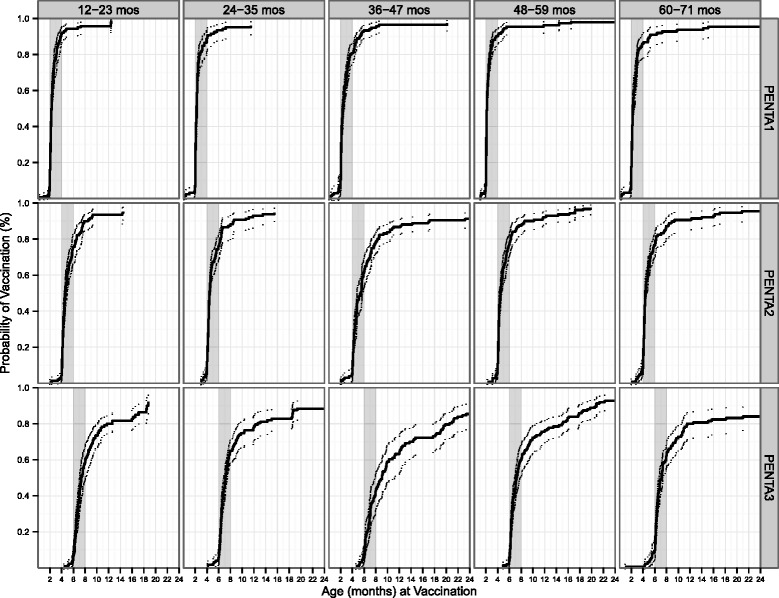
Time to vaccination, estimated as 1 – time to vaccination, and 95% point-wise CIs for all three doses of pentavalent vaccination among children in 5 age groups, Bolivar, Venezuela, 2011. Gray shaded area indicates the timeliness window for each dose (PENTA1: 2–4 months, PENTA2: 4–6 months, PENTA3: 6–8 months).

Approximately 90% of children received the first dose of pentavalent vaccine (Penta1) between 2 and 4 months of age (Figure 1); however, timeliness of subsequent doses decreased, even though coverage remained high. For example, most cohorts only achieved 50-70% coverage with Penta3 by the end of the recommended time of 6 to 8 months of age, although coverage by 8 months of age was only about 40% in the 36–47 month group. By 24 months of age, all 5 cohorts had reached 80-95% coverage with Penta3.

**Figure 2:**
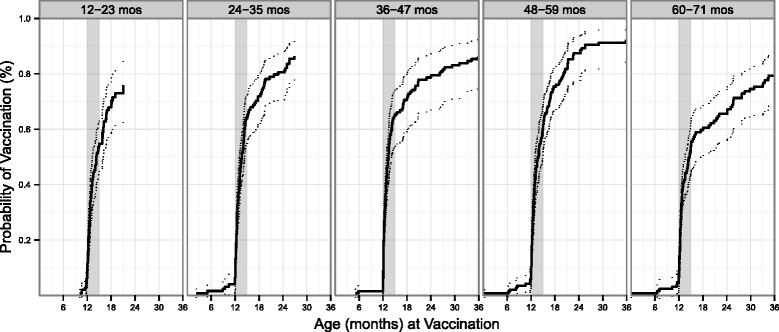
Time to vaccination, estimated as 1 – time to vaccination, for MMR1 vaccination among children in 5 age groups, Bolivar, Venezuela, 2011. Dotted lines represent 95% CI. Gray shaded area indicates the timeliness window (12–15 months) for the first dose of MMR.

There was more variability between cohorts in MMR1 coverage and timeliness of vaccination (Figure 2). The CI for the 12–23 month olds indicates coverage at 15 months was between 45 and 63%. The curves for the 23–35, 36–47, and 48–59 month groups indicate higher coverage by 15 months. MMR1 coverage gradually increased for all cohorts over the following two years.

## Discussion

Although one of VWA’s objectives in Bolivar in 2011 was to improve immunization coverage, only an estimated 24% of eligible children received at least one indicated vaccine dose and only 13% received all doses due. Overall, there was low awareness of VWA 2011; less than one-third of caregivers heard about recent vaccination activities, which may have limited the initiative’s reach. Nevertheless, vaccine coverage for most vaccines at the time of the survey was above 85%.

The proportion of children who benefitted from VWA 2011 was likely lower than our estimates indicate. Because VWA 2011 in Bolivar focused on increasing vaccination services in institutional settings that already provided them, we were unable to distinguish between vaccine doses provided because of VWA and those administered irrespective of VWA. In addition, the largest proportion of eligible children vaccinated were those who received BCG or birth dose of hepatitis B vaccine. However, these doses are typically administered following hospital births and 95% of births in Bolivar are estimated to occur in hospitals [11].

Although coverage with most vaccines was relatively high among all cohorts, MMR1 coverage among 12–23 month-olds was lower at 72% (95% CI 63-80%) than among older cohorts (88-93%). Analysis of timeliness demonstrated that older cohorts also had lower levels of coverage during the second year of life; however, continuing to offer immunization services to older children resulted in higher coverage during succeeding months and years, similar to findings reported elsewhere [12]. While the scope of this study did not address specific reasons for the receipt of late doses, particularly for Penta2 and Penta3, speculative reasons may include population movement across the border to Colombia for extended periods of time for employment, delaying the completion of schedules until families return to Venezuela, work obligations which limit the ability of parents to bring their children to the health centers during regular hours and the reduced use of house-to-house vaccination strategies. Such concerns were part of the reason that border municipalities were prioritized during VWA 2011 in Venezuela, as the initiative represents one strategy to identify those children with incomplete schedules and improve coverage.

Social mobilization to raise population awareness of public health interventions is essential [13-16], especially for institution-based efforts that require population turnout at health centers, as opposed to strategies such as house-to-house vaccination. Implementation of the VWA 2011 communication campaign was decentralized to the local level and had limited oversight from the national EPI. In the future, efforts to ensure messages about VWA reach a wide audience should use communication channels identified by caregivers in this evaluation, such as radio and health centers. While access to vaccination services was beyond the scope of this survey, it merits further examination, especially in a border municipality such as Bolivar which has high population mobility.

High prevalence of vaccination cards in households, high participation rate among caregivers aware of vaccination activities, and widespread use of public facilities for vaccination services indicate public support of EPI in Bolivar. Among children vaccinated, 45% did not receive all indicated vaccines. This may be due to multiple reasons, including those related to health care workers who may have been uncomfortable administering multiple vaccines, had misconceptions about contraindications, or were hesitant to open new vaccine vials for fear of wastage. There may have also been unknown vaccine and supply shortages and it is possible that parents may have been uncomfortable with their child receiving multiple vaccinations on the same day, among many other potential reasons [16,17]. To prevent such missed opportunities in the future, additional health worker training and assessments of parental satisfaction regarding their experience with the EPI should be considered.

Coverage for Penta3 in Bolivar was higher than the national coverage of 78% reported by Venezuela through the WHO/UNICEF Joint Reporting Form for 2011 [18]. Lower coverage for yellow fever vaccine, especially among children aged 12–23 months (63%) is likely due to global vaccine shortages, causing stock-outs. Rotavirus vaccine was introduced in Venezuela in 2006; because of this and the upper age limits for administering vaccine, older children would have aged-out of eligibility to receive the vaccine post-introduction, resulting in lower coverage in these cohorts. The schedule for rotavirus vaccination was modified in 2009, an operational change that could have also affected coverage. Other recent EPI schedule changes include the introduction of MMR2 and second OPV and DTP boosters during 2009–2010. These changes may explain the lower coverage for these doses as health workers adapted to new guidelines.

While our results exposed positive and negative aspects of implementation of VWA 2011 and the overall EPI in Bolivar, Venezuelan health authorities have used lessons learned for planning of subsequent vaccination activities. For example, efforts to improve VWA social communication and mobilization intensified in Bolivar in 2012, 2013 and 2014, including new strategies such as using loudspeakers. To optimize performance of routine EPI, local health authorities have subdivided parishes of the municipality and reassigned health workers to better reach the population. Health authorities have also coordinated with community councils to conduct house-to-house vaccination in specific difficult-to-reach and low coverage areas. An additional vaccination post was also opened in a hospital in Bolivar with extended evening hours to increase opportunities for working parents to have their children vaccinated.

The survey in Bolivar had several limitations. The survey was completed by multiple teams during an intensive two-day effort over a weekend when most parents are home. This decision was made to complete the survey quickly in order to maximize the efficiency of the data collection process. The use of multiple teams may have increased the risk of bias, but efforts were in place to train and closely supervise the interviewers. The survey design was based on a quota sample of households with at least one eligible child and not probability sampling. As a result, we could not calculate the selection probability of households or account for non-response. To accurately determine VWA 2011 eligibility, we restricted analysis to children with available vaccination records. This may have led to overestimation of coverage; however, because card availability was high overall within each age group, this bias was likely minimal.

## Conclusion

Our evaluation provided important information regarding the implementation and outcome of VWA 2011 in one border municipality, and provided insight into the performance of EPI in this challenging and dynamic area. Because of the targeted nature of the survey, findings cannot be generalized to VWA 2011 results in other areas of the country, nor do they assess all components of Venezuela’s multi-faceted VWA 2011 campaign. As Vaccination Weeks evolve within the framework of World Immunization Week, similar surveys, adapted to local realities, can help objectively assess execution and outcomes of similar initiatives aimed at improving coverage and updating childhood vaccination schedules, while providing lessons to adjust strategies and optimize results. Such surveys also serve as windows into routine EPI implementation at the local level, and are thus important management tools. Constructive activities following survey results, such as Venezuela’s response since 2011, will help ensure that populations are protected against VPDs and that disease eradication, elimination and control goals are achieved and maintained.

## Endnotes

^a^Bolivia, Chile, Colombia, Ecuador, Peru, and Venezuela

^b^Included nurses, “simplified medicine” assistants, and social workers;

^c^Pentavalent vaccine contains diphtheria toxoid, tetanus toxoids, pertussis vaccine, *Haemophilus influenzae* type b (Hib) vaccine, and hepatitis B vaccine.

## Abbreviations

BCG: Bacillus Calmette-Guérin vaccine
CI: Confidence Intervals
DTP: Diphtheria-tetanus-pertussis vaccine
EPI: Expanded Program on Immunization
MMR: Measles-mumps-rubella vaccine
MOH: Ministry of Health
OPV: Oral polio vaccine
PAHO: Pan American Health Organization
Penta: Pentavalent vaccine
Td: Tetanus and diphtheria toxoids
CDC: United States Centers for Disease Control and Prevention
VWA: Vaccination Week in the Americas
VPDs: Vaccine-preventable diseases
WHO: World Health Organization
